# Consumer electronics based smart technologies for enhanced terahertz healthcare having an integration of split learning with medical imaging

**DOI:** 10.1038/s41598-024-58741-0

**Published:** 2024-05-06

**Authors:** Sambit Satpathy, Osamah Ibrahim Khalaf, Dhirendra Kumar Shukla, Sameer Algburi, Habib Hamam

**Affiliations:** 1 CSE, Galgotias College of Engineering and Technology, Greater Noida, Uttar Pradesh India; 2 Department of Solar, Al-Nahrain Research Center for Renewable Energy, Al-Nahrain University, Jadriya, Baghdad, Iraq; 3 CSE, Galgotias University, Greater Noida, Uttar Pradesh India; 4 Al-Kitab University, 36015 Kirkuk, Iraq; 5 Uni de Moncton, Moncton, NB 1EA 3E9 Canada; 6 Hodmas University College, Taleh Area, Mogadishu, Somalia; 7 Bridges for Academic Excellence, Tunis, Centre-Ville, Tunisia; 8 School of Electrical Engineering, University of Johannesburg, Johannesburg, South Africa

**Keywords:** Medical imaging, Terahertz technology, Consumer electronics (CE), Split learning, Smart healthcare system, Energy science and technology, Engineering

## Abstract

The proposed work contains three major contribution, such as smart data collection, optimized training algorithm and integrating Bayesian approach with split learning to make privacy of the patent data. By integrating consumer electronics device such as wearable devices, and the Internet of Things (IoT) taking THz image, perform EM algorithm as training, used newly proposed slit learning method the technology promises enhanced imaging depth and improved tissue contrast, thereby enabling early and accurate disease detection the breast cancer disease. In our hybrid algorithm, the breast cancer model achieves an accuracy of 97.5 percent over 100 epochs, surpassing the less accurate old models which required a higher number of epochs, such as 165.

## Introduction

Breast cancer for women is very common disease in this world so that many techniques and algorithms are there to facilitating early cancer detection and diagnosis. To consider this, here it has been introduced smart consumer electronics-based data collection through IoT based system or wearable sensor. Secondly considering Terahz medical image as THz imaging is non-invasive, meaning it doesn’t require tissue samples or ionizing radiation like X-rays. This mitigates patient discomfort and eliminates the risks associated with invasive diagnostic procedures and having high clarity. For the machine learning model in training purpose EM algorithm is used, which optimized the input parameter. After training the Bayesian approach split learning model is used for early breast cancer detection with secure the patient data.

The whole article is organized as follows: Section one serves as the introduction, followed by the second and third sections, which consist of prior work and the problem definition, respectively. The fourth section outlines the proposed method, while the fifth section provides an in-depth description of the split learning-based algorithm designed for breast cancer prediction. The sixth module encompasses both the presentation of results and the subsequent discussion. Lastly, the paper concludes with a final module dedicated to summarizing key insights and findings. This is the way in which the whole article is get structured.

## Prior literature review

The examination of state-of-the-art thermal sensors, akin to those utilized in nocturnal vision cameras, has attracted significant interest. The groundbreaking advancements in semiconductor processing techniques and the development of innovative materials have unveiled new horizons in infrared (thermal) detectors^1^. Terahertz (THz) technology, which functions within the frequency range of 0.3 to 3 THz, harnesses thermal radiation's power and presents notable benefits across diverse domains^2,3^. In contrast to shorter wavelengths (millimeter waves) that encounter difficulties in penetrating materials, THz waves possess the capability to deeply penetrate matter, facilitating high-resolution imaging. Additionally, THz waves demonstrate characteristics such as non-ionization and low photon energies, ensuring that they do not elicit harmful reactions in tissues^4^.

The utilization possibilities of THz technology are broad and encompass swift mobile and wireless communication, environmental surveillance, safeguarding, and monitoring for the detection of hazardous substances and explosive materials, medical and healthcare uses, pharmaceutical delivery, T-ray imaging, material analysis, and even the remote identification of celestial substances and planets^5,6^. Despite notable advancements in THz technology post-2010, notably in high-powered sources, uncooled detectors, and real-time imaging, there exist underlying challenges that must be resolved to render these systems commercially feasible and seamlessly integrated into flexible electronics. The current trajectory of THz research leans towards an interdisciplinary approach driven by the practical needs of everyday commercial systems. This underscores the significance of examining innovative and robust Terahertz sources, responsive sensors, and efficient optical elements^7–10^. This study comprehensively explores the evolving trends in THz systems at the crossroads of artificial intelligence (AI) and various consumer electronic devices such as wearables and the Internet of Things (IoT). Split learning can be a valuable technique for privacy-preserving medical imaging applications. It permits healthcare establishments and scientists to cooperate and instruct deep learning models on medicinal image data, all the while upholding the decentralization and security of the unprocessed data. The significance of split learning extends to various aspects of medical data, including radiology and diagnosis, data privacy compliance, personalized medicine, remote diagnosis, and telemedicine^11,12^.

Emerging technologies within the healthcare sector, such as Artificial Intelligence (AI), Internet of Things (IoT), blockchain, and big data, play a pivotal role in the transformation of healthcare systems and enhancement of patient care. These technologies are utilized for a multitude of purposes, encompassing pandemic control, surveillance, contact tracing, as well as the diagnosis, treatment, and prevention of diseases like COVID-19. Moreover, they facilitate the creation of intelligent healthcare solutions that empower individuals to take charge of their own health and receive precise and efficient treatment and diagnosis^13,14^. Furthermore, the emergence of these technologies contributes to the analysis and enhancement of healthcare procedures, thereby resulting in improved healthcare outcomes. The potential for a transformative impact on healthcare delivery^15^, service quality improvement, and cost reduction exists within the integration of these technologies, thereby resulting in the ultimate transformation of society. Numerous researchers have utilized split learning, a distributed machine learning technique, within the medical domain^16,17^. This This methodology is aimed at addressing the limited availability of annotated data and the challenges linked to openly exchanging patient data. A comparison has been drawn between this approach and centrally hosted, non-collaborative setups for two specific medical deep learning assignments. Findings indicate that the performance of split learning remains consistent regardless of the number of clients involved, showcasing its advantages in collaborative training of deep neural networks within the healthcare sector. Furthermore, a Split Artificial Intelligence Architecture (SAIA) has been introduced for mobile healthcare systems, blending cloud computing infrastructure with lightweight AI solutions that operate locally on the client side. SAIA consistently surpassed its benchmarks in terms of both effectiveness and efficiency^18–21^. These strategies underscore the promise of distributed learning and split AI architectures in enhancing healthcare outcomes. Terra hertz technology has had a significant impact on the healthcare system, as extensively explored by numerous researchers and scientists. Advanced terahertz healthcare systems strive to boost medication adherence, diagnosis, and treatment efficacy through the utilization of multimedia technology and remote monitoring^22–26^. These systems encompass a variety of components such as medicine bags, home servers, hospital servers, prescription terminals, and pharmacy servers, thereby streamlining communication and data exchange^27,28^.

The incorporation of wireless communication technology enables prompt evaluation of effectiveness and comprehensive compilation of health records^29,30^. The utilization of terahertz technology has demonstrated promise in various medical contexts such as identifying skin cancer, detecting dental caries, and screening pharmaceuticals. Nevertheless, there exist constraints and obstacles that require resolution prior to the extensive implementation of terahertz technology in clinical environments.. The harmless and efficient nature of terahertz range, along with its distinctive properties and biophysical consequences, have been substantiated in medical devices^31,32^. As a whole, the prospective implementation of advanced terahertz healthcare systems holds the capacity to ameliorate healthcare outcomes and enhance patient experiences.

## Research gap and problem definition

The amalgamation of artificial intelligence (AI) and intelligent terahertz technology presents an intriguing frontier in the realm of consumer electronics, particularly within healthcare applications^33–36^. By combining AI's cognitive capabilities with terahertz's proficiency in non-invasive imaging, a groundbreaking healthcare system emerges as a plausible prospect. This fusion equips consumer electronic devices with the capacity to conduct advanced medical scans, allowing users to oversee their well-being in unprecedented ways. Ranging from the identification of early-stage irregularities to the monitoring of chronic conditions, this advancement ensures personalized insights and timely interventions, all conveniently accessible from one's residence^37–40^. The inclusion of AI enriches the diagnostic precision and interpretation of this system, rendering it user-friendly and dependable. The potential for wearables, smartphones, and other consumer electronics to function as health monitoring tools signifies a paradigmatic shift in proactive healthcare. By enhancing the accessibility and viability of healthcare data, individuals are empowered to take charge of their health in an unparalleled manner. Essentially, the AI-driven intelligent terahertz healthcare system tailored for consumer electronics envisions a future where empowerment and well-being seamlessly converge.

The research gap is evident in two significant aspects, namely, the effective utilization of split learning for breast cancer prediction while incorporating data obtained from consumer wearable devices. The challenges that arise encompass the collection of data and the integration of the Internet of Things (IoT) and wearable smart consumer electronics (CE) devices for health monitoring and sensor purposes. These devices possess the capability to promptly accumulate patient/consumer information, including vital signs such as heart rate and temperature, in real-time and subsequently transmit this data to the healthcare system for analysis. Furthermore, the subsequent step entails the development of a novel model that integrates split learning with terahertz medical imaging for the early detection of breast cancer. This model is characterized by its high throughput and secure technology ^41–46^.

## Proposed method

The entirety of the proposed model’s architecture is composed of three distinct components. The initial component entails data collection, which is executed via the utilization of smart consumer electronics-based data collection employing an IoT-based system or a wearable sensor. The subsequent phase entails the provision of guidance and the subsequent evaluation and validation of the outcomes of the model. With regard to instruction, the EM optimized algorithm has been utilized, while the Bayesian-based split learning model has been employed for the anticipation of breast cancer.

Preprocessing for Data Analysis and Materials/Methods in the context of using terahertz (THz) imaging for breast can- cer identification involves several key steps: such as data collection and acquisition, preprocessing of data, feature extraction, and splitting.

### Data acquisition

**Figure 1:**
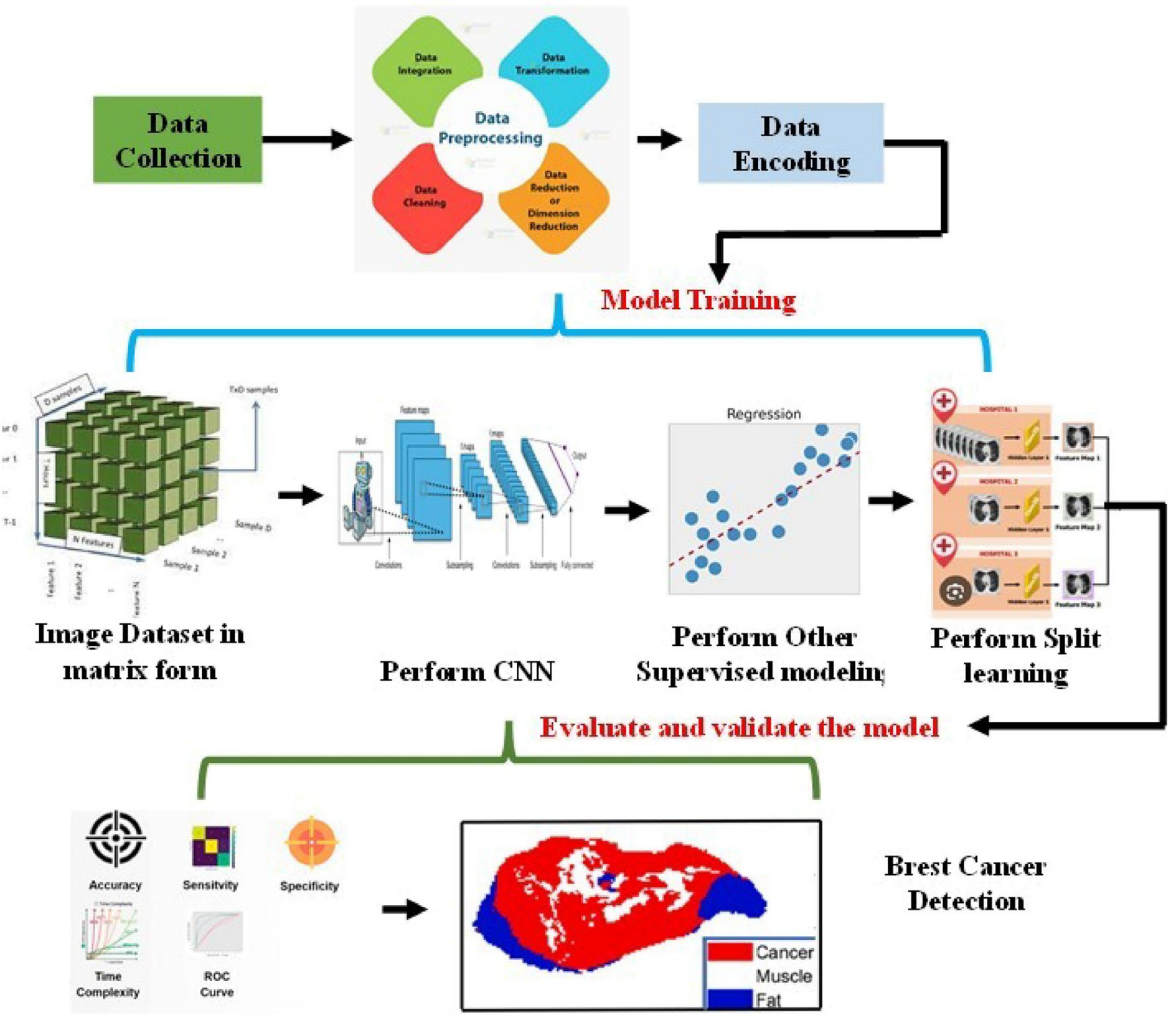
Architecture of newly proposed healthcare prediction system using split learning method.

**Figure 2:**
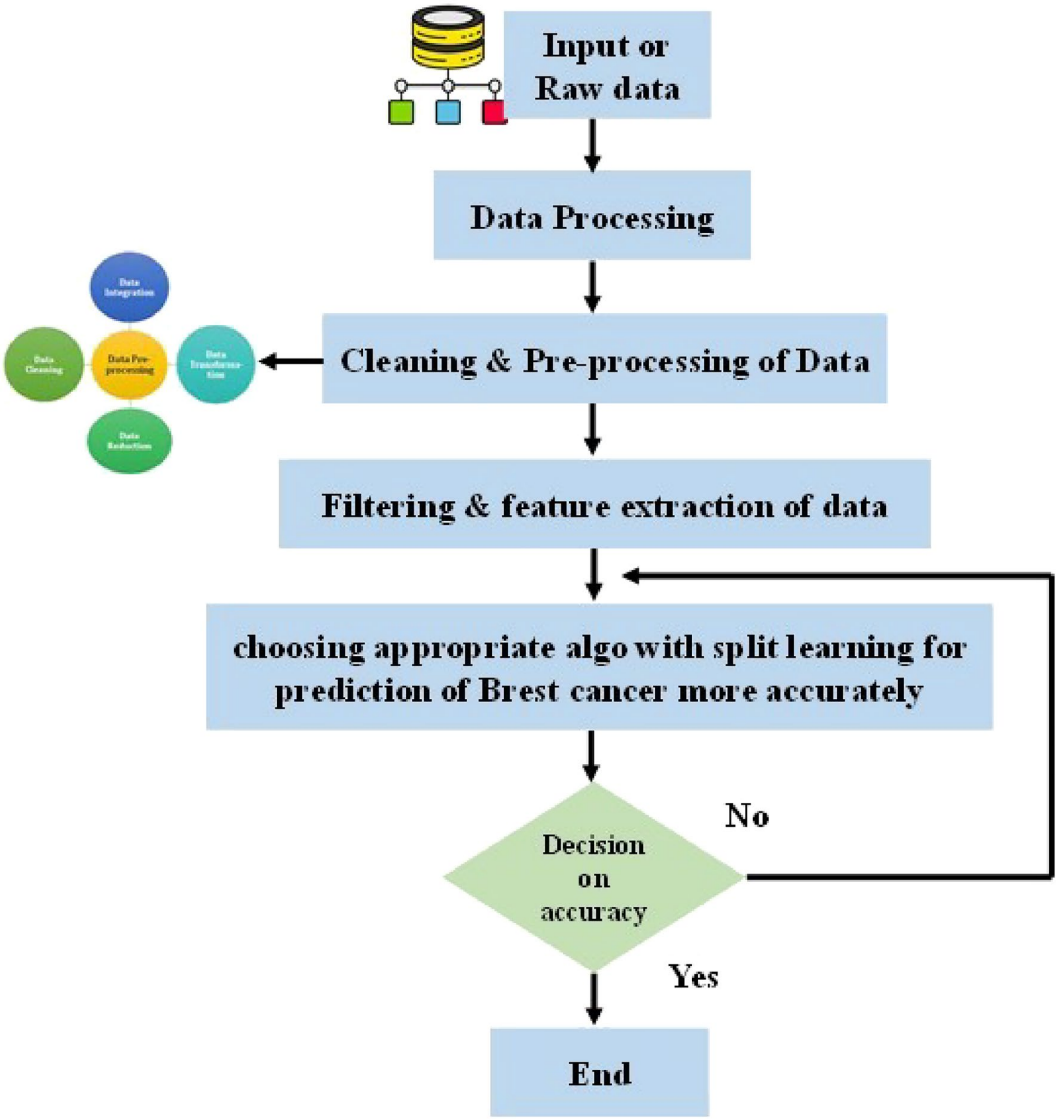
Step by step process from collection of data to choosing appropriate model with split learning for predict the health- care condition, the whole method.

In Data Acquisition, the gather THz imaging data from recently removed mouse tumors, ensuring that the data is accurately recorded and properly labeled for breast cancer identification. This module is employed for the purpose of eliminating any form of noise or artifacts that may be present in the THz data, thereby guaranteeing that the dataset is devoid of any anomalies that might have a detrimental impact on the performance of the model. At present, there exists a necessity to identify a suitable feature, a requirement that is met by the feature extraction method. Consequently, it becomes imperative to ascertain the pertinent features or characteristics from the THz images, which can subsequently be utilized as input variables for the models. This may involve techniques such as edge detection or texture analysis. In the very next level, it has been dividing the dataset into training and testing sets to assess model performance. Given that the approach aims to reduce the demand for extensive training data, this splitting should be designed to optimize data uses (see Figs. 1 and 2).

The mathematical equation for data acquisition can be represented using the concept of sampling. Data acquisition typically involves sampling a continuous signal at discrete time intervals. The equation for data acquisition is often expressed as:

1 $$x[n] \, = x(t_{n} ) \, = f(nT_{s} )$$

where x[n] is the sampled data at the discrete time index n, x(t_*n*_) represent the continuous signal x(t), sampled at a specific time t_*n*_, F(nT_*S*_) is the function that samples the continuous signal x(t) at discrete time interval. “n” is the index of the discrete samples; T_*S*_ is the sample interval. This equation describes how continuous data is converted into a discrete form through sampling. The choice of the sampling interval T_*S*_ is critical and is determined by the Nyquist-Shannon sampling theorem, which states that T_*S*_ should be less than or equal to half the reciprocal of the highest frequency component present in the signal to avoid aliasing.

### Preprocessing of the raw data

In this section, it has been discussing the pre-processing method, which contain from sampled data to normalized, scaled etc. Once it has been getting the data, the preprocessing encompasses various steps such as normalization, imputation, and scaling. The whole process is shown in Fig. 2, which started from raw data to the choosing an appropriate model. The mathematical equation for a common preprocessing step, min–max normalization, can be represented as:

2 $$X_{normalized} = \frac{{{\text{x}} - {\text{min}}(x)}}{maz(x) - min(x)}$$

where x is the actual/ original data point, X_*normalized*_ is the normalized data point, min(x) and max(x) are the minimum and maximum value in the data set. After getting the normalized value filter is used to remove the unwanted data from the signal. Here we have to choose which type of filter is used, it may be a low pass or high pass filter as per the requirement. Here a simple mathematical representation of a low pass filter is given

3 $$Y(t) = \int {x \cdot t^{t} \sum h(t - t^{t} )} dt^{t}$$

where, y(t) is the filter signal, x(t) is the original signal, h(t) is the impulse response of the filter signal.

### Feature extraction

Once the scaled data is obtained, it becomes necessary to identify the suitable feature from this data. Due to the presence of numerous parameters in the data, the PCA method is employed to identify the relevant data that can aid in predicting the healthcare status. In the subsequent discussion, we will elaborate on the step-by-step process of PCA for feature extraction and which is shown in Fig. 1. This figure tells detail about starting from choosing of raw data, filter process, feature extraction (PCA method), and finally considering appropriate training, testing and validation model.

After the completion of the filtering stage, it is necessary to carry out the process of feature extraction. The purpose of feature extraction is to convert the data into a collection of pertinent features. Principal Component Analysis (PCA) is a widely used method for reducing the dimensionality of the data and extracting features. The mathematical formula for PCA involves the identification of the principal components through eigen decomposition.

4 $$X = W \cdot Z$$

where X is the original data, which represented as in the matrix form. W is the is the principal component (matrix of eigenvectors). Z is the is the transformed data having reduced dimensionality and represented in the form of matrix. PCA seeks to find W such that the transformed data Z retain the X-dimensionality. For the next step it has consider X as input data.

### Implement with appropriate algorithm with split learning

In this section, we have amalgamated the split learning technique with the machine learning model to facilitate the early identification of breast cancer through the utilization of terahertz medical imaging. Within the machine learning model, we have employed the Bayesian approach to forecast the occurrence of breast cancer. The incorporation of split learning methodology ensures the privacy of sensitive and confidential patient information. Subsequently, an elaborate explanation of the newly proposed mathematical model and the intricate details of the split learning-based hybrid algorithm will be discussed below.

A newly proposed model, integrating with split learning for early detection breast cancer using terahertz medical imaging.

Training the machine learning model is of utmost importance to predict breast cancer accurately. The model’s accuracy is heavily dependent on the quality of the training data. In this study, FFPE samples^22^ were used as a reference point for the training purposes. However, the dehydration process can negatively impact the quality of the training data due to differences in shape between freshly excised samples and their FFPE counterparts. To overcome this challenge, we propose an innovative approach, such as the EM algorithm, which aims to select training data based on reliability.

Once we train the machine learning model, now we can used the appropriate algorithm to predict the breast cancer. For the model terahertz (THz) imaging of the given mouse data is act as input. And we can used this data to the proposed supervised multinomial Bayesian with split learning for the identification of breast cancer. One more advantages of this model is, it requires only a small set of model parameters compare to other proposed models^22^. Detail of the mathematical model of the proposed algorithm can see module.Algorithm: Hybrid Training Procedure using split learning.

**Figure:**
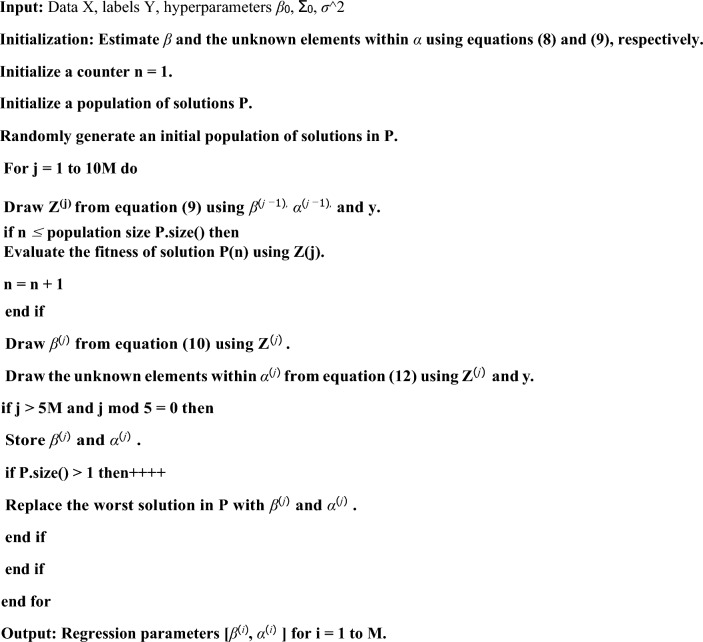
Algorithm: Hybrid Training Procedure using split learning.

### Development of split learning model

Mathematically formulating the split learning algorithm for predicting healthcare diseases necessitates the articulation of the fundamental elements and procedures of the algorithm in mathematical symbols. Here it has lies a comprehensive mathematical representation of the split learning procedure:

As lastly, we have obtained the featured input data X, which is represented in a matrix of size n × m, where n is the number of samples (patients/ mouse) and m is the number of features (attributes/ parameters). Let Y represent the predicted value as output for the given input data X. So that the input X, in the form of function F_*c*_ defined as

5 $$F_{c} = (X;\theta_{c} )$$

where F_*c*_ represents the input model function with parameters *θ*_c_. This model extracts features from the input data.

Similarly the F_*s*_ the output function parameters *θ*_s_ can defined as

6 $$F_{s} = (F_{c} (X;\theta c);\theta_{s} )$$

This model makes the final disease predictions based on the features extracted by the input model. The split learning process involves iterative communication rounds between the input and output models until convergence: In each iteration, the input model updates its features using the following equation:

7 $$X^{j} = Fc(X;\theta_{c} )$$

The output model takes these features and makes predictions:

8 $$Y^{t} = Fs(X^{t} ;\theta s )$$

The input model updates its parameters using the predictions and a loss function

9 $$\theta c \leftarrow \theta c - \alpha \nabla \theta cL(Y^{t} ,Y)$$

Similarly the output model also updates its parameters using the same loss function:

10 $$\theta s \leftarrow \theta s - \alpha \nabla \theta sL(Y^{t} ,Y)$$

This process continues until convergence, where *α* is the learning rate. After achieving convergence, the ultimate pa- rameters of the global model are acquired through the process of the parameters of the model at the input and output function. Appropriate evaluation metrics can be utilized to evaluate the efficiency of the model, encompassing accuracy, precision, recall, and F1-score, amongst others. The aforementioned mathematical modeling offers a comprehensive framework for comprehending the split learning algorithm employed in healthcare disease prediction.

### Testing process in the proposed model

In order to determine whether there is a significant difference between the average values of cancerous and non-cancerous pixels within each test sample using our proposed model. The first step was to conduct a univariate t-test. This analysis used the first component of the low-dimensional vector per pixel, which was obtained from the output of the proposed algorithm.

The null hypothesis of this t-test assumes that there are equal means between the outputs of cancerous and non-cancerous pixels. Table 1 presents the results of the t-test, which showed that the p-values for all test samples were almost zero. This indicates that the null hypothesis can be rejected, confirming that there are significant differences in the mean values of the outputs of cancerous and non-cancerous pixels, as revealed by the t-test results.

**Table 1:** Three different data sett-test result with the significance value is 0.05.

Various samples	Degree of freedom	Standard deviation	S-test	P-value	Confidence interval
Mouse 13A fresh	202	0.4101	32.5432	4.9622 × 10^*−*76^	[1.8111, 1.9877]
Mouse 10B fresh	202	0.7233	19.7843	7.221 × 10^*−*43^	[1.7110, 2.200]
Mouse 9B fresh	202	0.6422	20.2006	1.551 × 10^*−*41^	[1.5992, 1.9675]

## Result

To confirm the substantial reduction in computational complexity achieved by the proposed algorithm during the training procedure, we conducted a comparative analysis between the results obtained from the proposed classifiers and other ones.

The results, as presented in Tables 1 and 2, clearly demonstrate about the choosing sample and finding out the area under cover using various model with our proposed model respectively. In order to be considered the THz image, only the tissue during its histopathology process has been taken into consideration. We have proposed an EM-algorithm for the method of data selection in training, and only data that surpass a certain threshold of reliability are utilized for the training. This allows us to use only a few parameters for our training process.

**Table 2:** Comparison of our proposed mode with other various model for the mouse 9B fresh data, considering area under the ROC curve.

Region	1D MCMC	2D EM	3D polynomial regression	3D supervised kernel method	Proposed split learning model
Fat	0.787	0.906	0.914	0.915	0.917
Muscle	0.77	0.713	0.861	0.868	0.868
Cancer	0.865	0.906	0.926	0.926	0.928

The experimental findings demonstrate that the recommended supervised regression models surpass current algorithms, including 1D MCMC and 2D EM, in all areas of concern. For instance, when employing the supervised polynomial regression approach, the areas under the ROC curves for cancer and muscle in Mouse 9B fresh increase from 90.68% to 92.71% and 71.35% to 86.18%, respectively.

## Discussion

Concerning the muscle and fat region, it is important to note that the proposed supervised segmentation models achieve a significant increase in area when compared to their unsupervised counterparts, going from 69.70%–72.63% to 75.25%–86.18%^44–46^. These results represent a step forward in achieving optimal differentiation between cancerous and non-cancerous tissue within freshly excised BCS. However, in our proposed split learning model, it has been observed that the highest areas under the ROC curves are obtained among all the presented classifiers for all three categories. This illustrates the high efficiency and optimization of our model in terms of time.

### Analysis of given model

**Figure 3:**
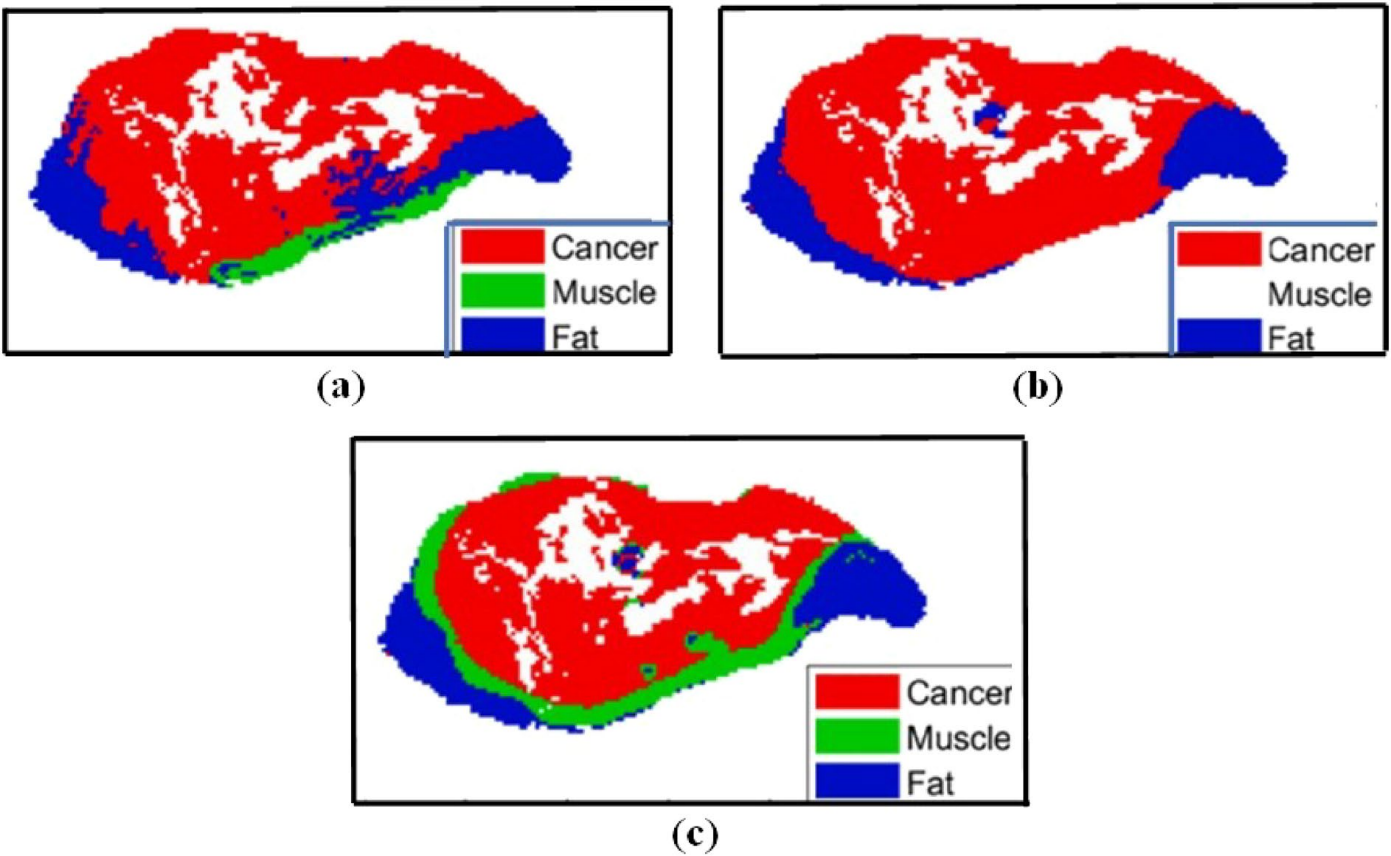
THz image of a Sample mouse sample data for cancer, muscle and fat.

**Figure 4:**
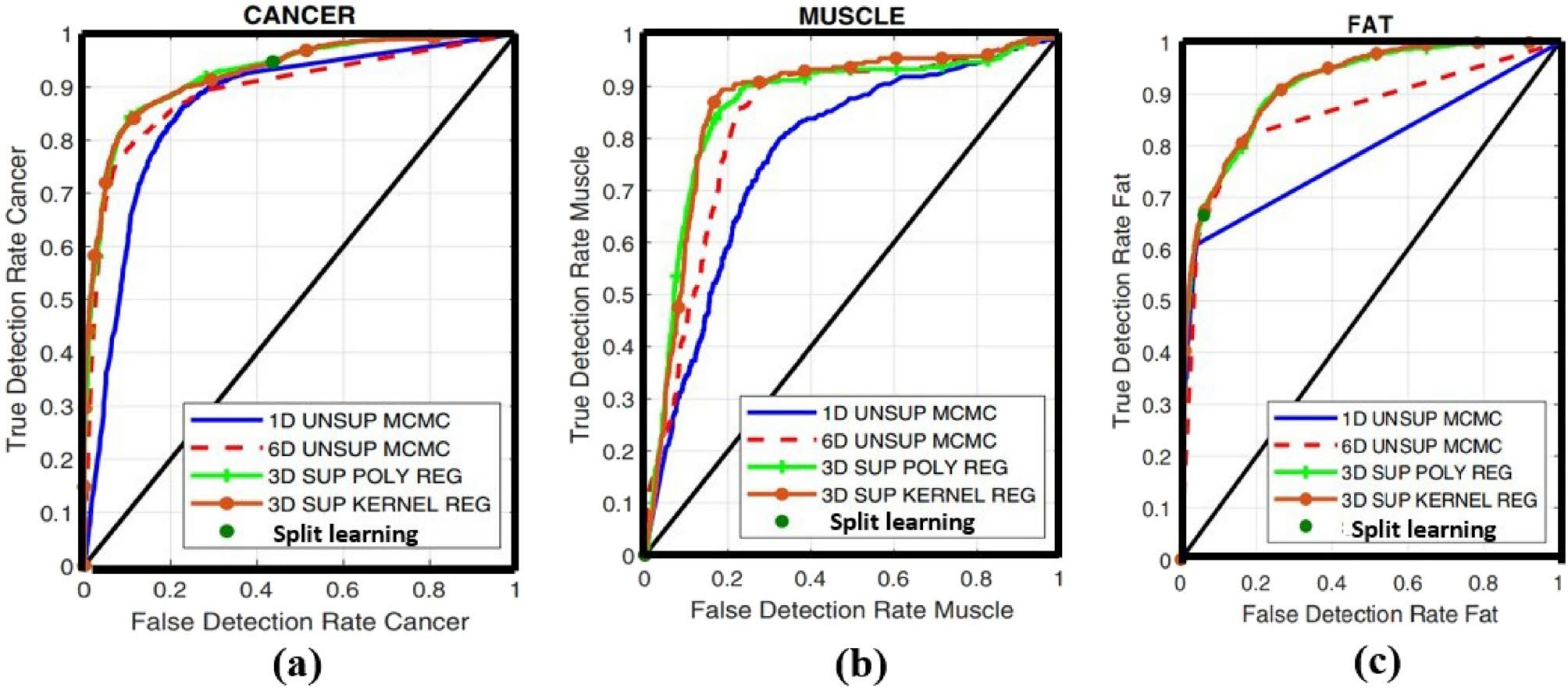
Comparison of the proposed split learning model with others having different samples.

**Figure 5:**
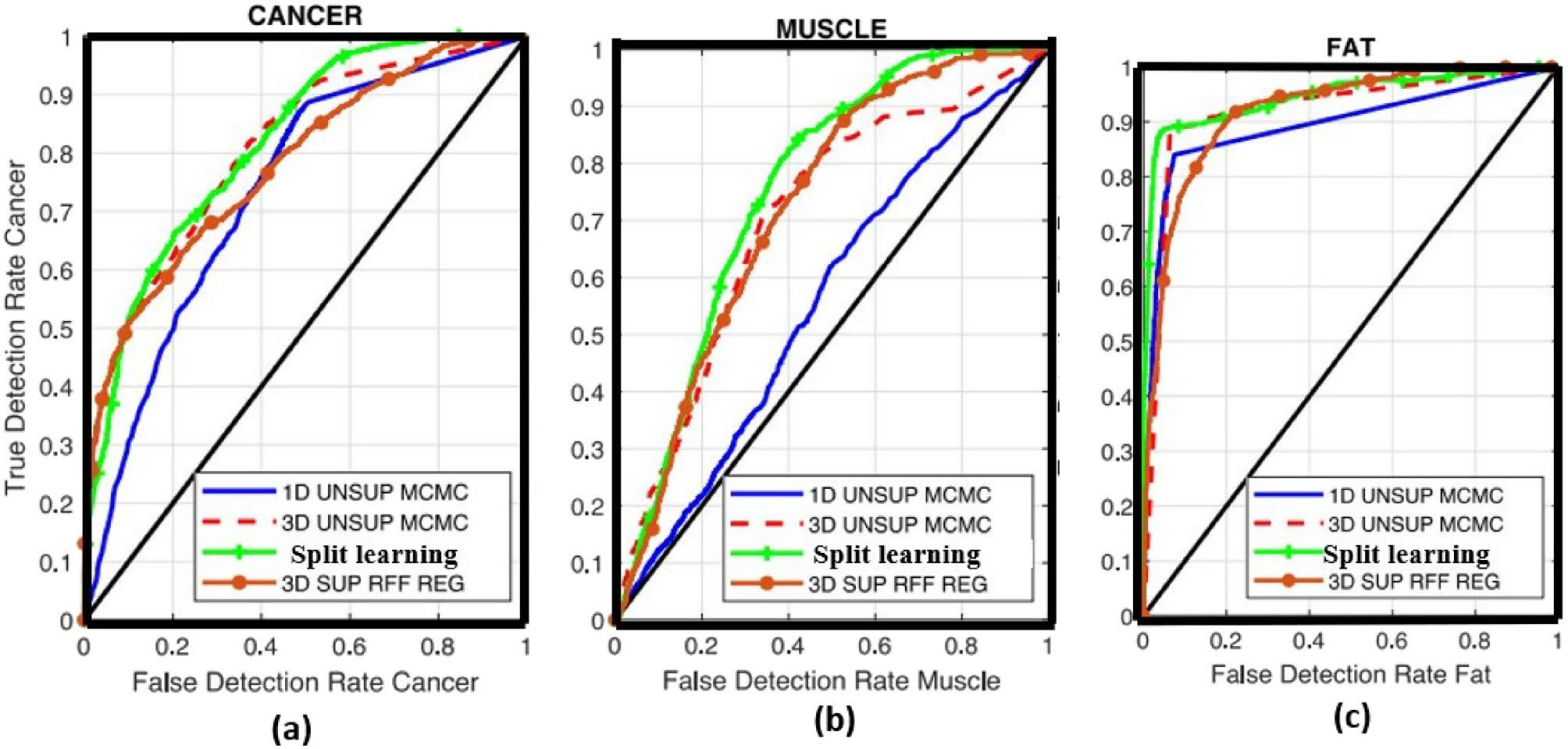
Comparison of the split learning model with few other examples (**a**) Cancer, (**b**) Muscle, (**c**) FAT.

The segmentation outcomes produced by the Bayesian model with the split learning are subsequently High value of ROC then other proposed model such as 3D polynomial regression, Supervised kernel method with those of the proposed kernel regression classifier, 1D MCMC and 2D EM model which is shown in Figs. 3, 4 and 5 (simulated by matlab version 2022a). Specifically, it becomes evident that while the split learning approach exhibits potential in detecting cancer and fat regions, it falls short in completely identifying the muscle region, as depicted in Figs. 4 and 5.

Figure 3 has represented the Terahz image of the sample is fresh and cancer. In Figs. 4 and 5 contain the information for 3 region such as cancer, muscle and fat. In this figure, it has been found fitted model with different algorithms with our proposed mode having the split learning. It is imperative to acknowledge that the results of these models were obtained through the utilization of the most favorable segmentation thresholds derived from each Receiver Operating Characteristic (ROC) curve. This approach emphasized the identification of cancer in all areas, with muscle or fat being the subsequent priority. The segmentation outcomes of the split learning model suggested in this research are subsequently compared to those of the SVM, kernel regression classifier in Figs. 4 and 5.

Split learning, a form of distributed machine learning, improves both accuracy and security by distributing the training of the model among different entities without the need to share raw data. In contrast to traditional techniques such as SVM and kernel methods, split learning enables advanced model training on decentralized data, ensuring privacy and minimizing the possibility of data breaches. This approach is particularly more effective in scenarios where data cannot be centralized due to privacy regulations or logistical issues, leading to improved model performance and robustness while maintaining data confidentiality.

### Statistical validation of the proposed model

**Figure 6:**
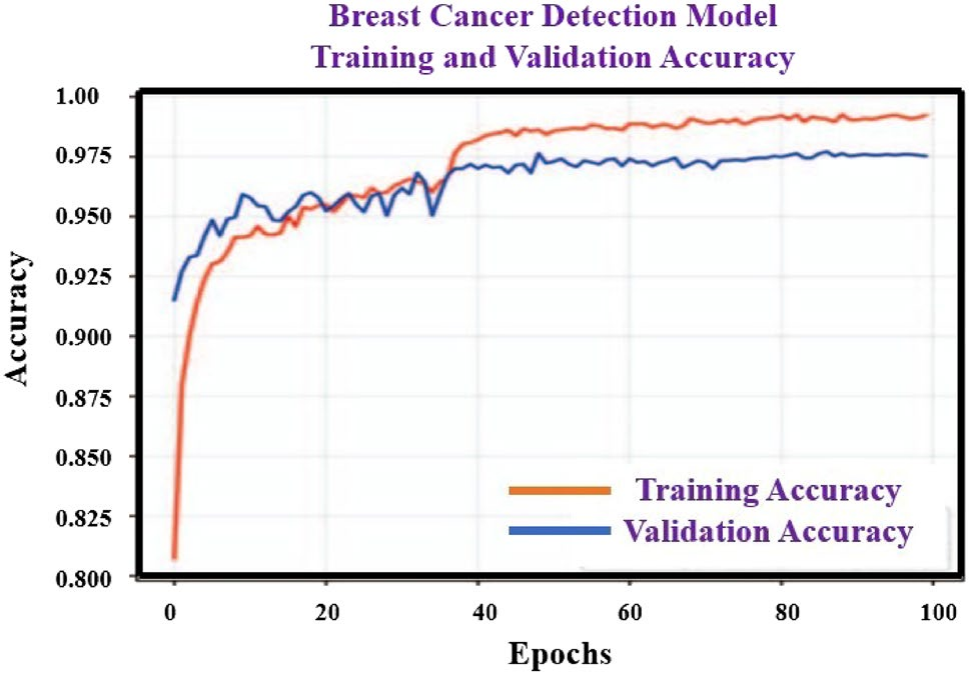
Considering the breast cancer data set and using the proposed split learning based model to find training and validation accuracy.

**Figure 7:**
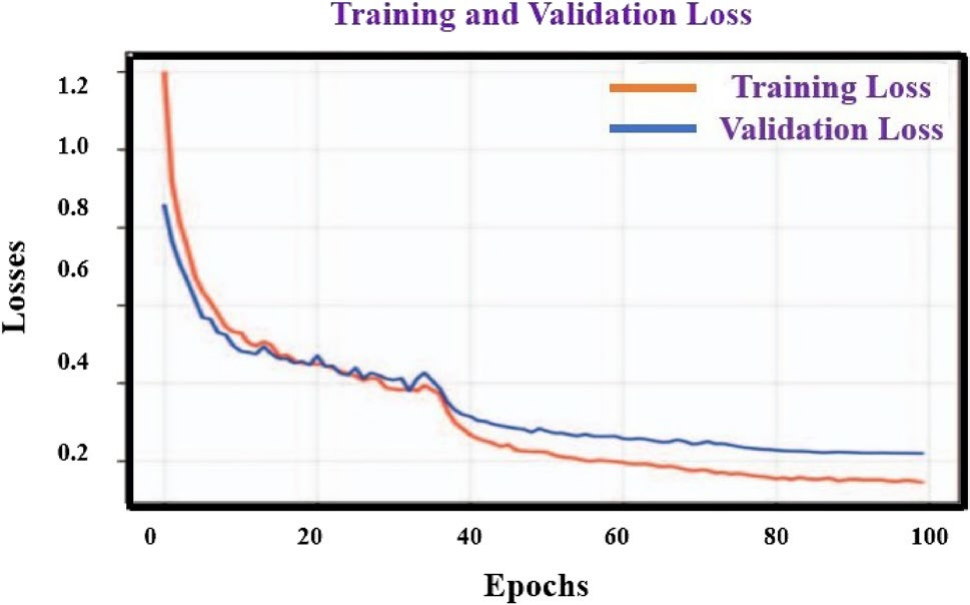
Considering the breast cancer data set and using the proposed split learning based model to find training and validation loss.

Statistical verification of breast identification and the suggested models is comprehensively explicated in Table 3, in that order, accompanied by corresponding learning curves depicted in Figs. 6 and 7. The training of the breast model was concluded after 100 epochs, attaining a plateau of 97.5% precision on the validation dataset. As for the other model (excluding the suggested model), training was terminated after 145 epochs, with the highest accuracy for the validation dataset transpiring during the 69th epoch. Consequently, the condition of the model at that juncture was employed for the purpose of analysis. After undergoing training, validation was conducted on both the breast and proposed models using real-world datasets. Accuracy, precision, recall, and F1-score for each class in the breast and proposed models are presented in Table 3.

**Table 3:** Accuracy, precision, recall, and the F1-score of the breast detection for proposed model evaluated on the real-world data.

8 various samples	Accuracy	Precision	Recall	F1-score
Breast cancer	0.97	0.93	0.96	0.95
No breast cancer	0.93	0.97	0.93	0.95
Implants	1.00	1.00	0.99	1.00

We have calculate parameters such as True Positives, True negative, false positive and false negative which are called TP, TN, FP, and FN respectively. In this work, we have assumed that to considered TP as “*µ*”, TN as “*µ*_1_” and FP as “Γ”, FN as “Γ_1_”. Based on this value, it can be find all the four parameters likely Accuracy, precision, recall, and the F1-score which helps to find the performance of our model for prediction of breast cancer using the split learning. So it can calculate the above parameters accuracy, precision, recall, and the F1-score respectively as shown in the Table 3.

The breast model exhibited an overall accuracy of 97.5%. It is noteworthy that only one image containing a breast was misclassified as belonging to the "no-breast" category, while two images without breasts were mistakenly categorized. Inter- estingly, the breast model displayed exceptional performance in identifying images with breast implants, achieving flawless recognition without any false assignments to this particular category in the test set.

## Conclusion

In addition, it has been introduced a hybrid Bayesian approach with split learning, which helps to find cancer using THz imaging of available data samples. This algorithm produces multinomial Bayesian ordinal probit regression models to conduct classifications within the THz images. To determine the connection between terahertz features and their corresponding classification outcomes, the method employs two different notable advancement. One is the unique thing of the model having very few training parameters and secondly the addition of the split learning the prototype is highly secured. Additionally, the use of split learning contributes to the improvement of the effectiveness and safety of collaborative medical data in the early detection of breast cancer by utilizing terahertz medical imaging. This has the potential to accelerate advancements in this critical area of healthcare.

Split learning integration with medical imaging improves the efficiency and accuracy of healthcare diagnosis and treatment. It enables collaborative training on decentralized data while maintaining data privacy, allowing neural network training for Brest cancer images without sharing patient data across hospitals. The proposed hybrid method uses a data quality-based adaptive averaging strategy to handle variations in annotated ground truth quality, ensuring accurate segmentation of given images. Additionally, EM training algorithm, SVM with split learning method overcomes performance drops from data heterogeneity, achieving comparable results toother algorithms such as SVM, kernel method. The training of the breast model was concluded after 100 epochs, attaining a plateau of 97.5% precision on the validation dataset. As for the other model (excluding the suggested model), training was terminated after 145 epochs, with the highest accuracy for the validation dataset transpiring during the 69th epoch. Consequently, the condition of the model at that juncture was employed for the purpose of analysis. After undergoing training, validation was conducted on both the breast and proposed models using real-world datasets. Moreover, these approaches demonstrate the suitability of split learning for collaborative learning in medical imaging and pave the way for future real-world implementations.

## Future work

Apart from these two advantages the smart consumer electronics devices like IoT devices, wearable health monitors and sensors have possessed the capability to amass patient information in real-time with other parameters to the healthcare system for analysis, in tandem with terahertz imaging. Also, in future we may integrated Cloud computing resources and servers are crucial for storing and processing large volumes of medical data and performing distributed split learning tasks.

## Data Availability

All data generated or analysed during this study are included in this published article.

## References

[1] Zhu M, Zhang J, Hua B, Lei M, Cai Y, Tian L, You X (2023). Ultra-wideband fiber-THz-fiber seamless integration communication system toward 6G: Architecture, key techniques, and testbed implementation. Sci. Chin. Inf. Sci..

[2] Huang Y, Shen Y, Wang J (2023). From terahertz imaging to terahertz wireless communications. Engineering.

[3] Chi, H. R., Domingues, M. D. F., Zhu, H., Li, C. & Kojima, K. Healthcare 5.0: In the perspective of consumer internet-of-things-based fog/cloud computing. in *IEEE Transactions on Consumer Electronics* (2023).

[4] Gezimati M, Singh G (2023). Advances in terahertz technology for cancer detection applications. Opt. Quant. Electron..

[5] Alkhabet MM, Ismail M (2023). Security algorithms for distributed storage system for E-health application over wireless body area network. J. Ambient Intell. Hum. Comput..

[6] Darwish, T. *et al.* Integration of advanced health technology within the healthcare system to fight the global pandemic: Current challenges and future opportunities. *Innov. Clin. Neurosci. *(2021). PMC8195559 34150361

[7] Mathanker SK, Weckler PR, Wang N (2013). Terahertz (THz) applications in food and agriculture: A review. Trans. ASABE.

[8] Chen X (2022). (THz) biophotonics technology: Instrumentation, techniques, and biomedical applications. Chem. Phys. Rev..

[9] Srivastava G, Agarwal S (2022). Terahertz imaging: Timeline and future prospects. Terahertz Dev. Circuits Syst. Mater. Methods Appl..

[10] Attaran M (2022). Blockchain technology in healthcare: Challenges and opportunities. Int. J. Healthc. Manag..

[11] Kim W, Kim J (2019). Innovative technologies for the smart E-Healthcare system. Investig. Clin. Urol..

[12] Maarten, G. *et al.* Split learning for collaborative deep learning in healthcare. *Learning* (2019).

[13] Zhuang, D., Nguyen, N., Chen, K. & Chang, J. M. Split artificial intelligence architecture for mobile healthcare system. *Arxiv* (2020).

[14] Xie J, Qian J, Wang T, Zhou L, Zang X, Chen L, Zhuang S (2023). Integrated terahertz vortex beam emitter for rotating target detection. Adv. Photon..

[15] Banerjee A, Vajandar S, Basu T (2020). Prospects in medical applications of terahertz waves. Terahertz Biomed. Healthc. Technol..

[16] Danciu TM (2019). Terahertz spectroscopy and imaging: A cutting-edge method for diagnosing digestive cancers. Materials.

[17] Zhang F, Tominaga K, Hayashi M, Tani M (2021). A quantitative interpretation for the difference of terahertz spectra of DL- and L-alanine: Origins of infrared intensities in terahertz spectroscopy. J. Phys. Chem. C.

[18] Badin, A. V. *et al.* Continuous wave THz imaging system for defectoscopy of polymeric ferroelectric materials. in *2022 IEEE 23rd International Conference of Young Professionals in Electron Devices and Materials (EDM)*, 618–623 (2022).

[19] Hu J (2022). Autonomous dynamic line-scan continuous-wave terahertz non-destructive inspection system combined with unsupervised exposure fusion. NDT E Int..

[20] Pan M (2022). Dielectric metalens for miniaturized imaging systems: progress and challenges. Light Sci. Appl..

[21] Gao S, Yang K, Shi H, Wang K, Bai J (2022). Review on panoramic imaging and its applications in scene understanding. IEEE Trans. Instrum. Meas..

[22] Chavez T, Vohra N, Bailey K, El-Shenawee M, Wu J (2021). Supervised Bayesian learning for breast cancer detection in terahertz imaging. Biomed. Signal Process. Control.

[23] Han C, Wu Y, Chen Z, Chen Y, Wang G (2024). THz ISAC: A physical-layer perspective of terahertz integrated sensing and communication. IEEE Commun. Mag..

[24] Lee G, Lee J, Park QH, Seo M (2022). Frontiers in terahertz imaging applications beyond absorption cross-section and diffraction limits. ACS Photon..

[25] Sun X (2023). Gradient-reduced graphene oxide aerogel with ultrabroadband absorption from microwave to terahertz bands. ACS Appl. Nano Mater..

[26] Mohammed NA, Khedr OE, El-Rabaie ESM, Khalaf AA (2023). High-sensitivity early detection biomedical sensor for tuberculosis with low losses in the terahertz regime based on photonic crystal fiber technology. Photon. Sens..

[27] Gavdush AA, Cherkasova OP, Tuchin VV (2019). The progress and perspectives of terahertz technology for diagnosis of neoplasms: A review. J. Opt..

[28] Vohra N, Liu H, Nelson AH, Bailey K, El-Shenawee M (2022). Hyper-spectral terahertz imaging and optical clearance for cancer classification in breast tumor surgical specimen. J. Med. Imaging.

[29] Yan Z, Zhu LG, Meng K, Huang W, Shi Q (2022). THz medical imaging: from in vitro to in vivo. Trends Biotechnol..

[30] Castro-Camus E, Koch M, Mittleman DM (2021). Recent advances in terahertz imaging: 1999 to. Appl. Phys. B.

[31] Gezimati M, Singh G (2023). Curved synthetic aperture radar for near-field terahertz imaging. IEEE Photon. J..

[32] Hu J (2022). Autonomous dynamic line-scan continuous-wave terahertz non-destructive inspection system combined with unsupervised exposure fusion. NDT E Int..

[33] Chhabra, C. & Sharma, M. Machine learning, deep learning and image processing for healthcare: A crux for detection and prediction of disease. In *Proceedings of Data Analytics and Management: ICDAM 2021*, Vol. 2, 305–325 (Springer, Singapore, 2022).

[34] Fabrizio MD (2021). Performance evaluation of a THz pulsed imaging system: Point spread function, broadband THz beam visualization and image reconstruction. Appl. Sci..

[35] Woodward RM (2002). Terahertz pulse imaging in reflection geometry of human skin cancer and skin tissue. Phys. Med. Biol..

[36] Berry E (2003). Do in vivo terahertz imaging systems comply with safety guidelines?. J. Laser Appl..

[37] Kakimi R, Fujita M, Nagai M, Ashida M, Nagatsuma T (2014). Capture of a terahertz wave in a photonic-crystal slab. Nat. Photon..

[38] Huang, Y., Shen, Y. & Wang, J. Terahertz imaging to terahertz wireless communications. *Engineering* (2022).

[39] Hu, B., Isaac, M., Majeed, A. P. A. & Liu, H. Edge intelligence-based e-health wireless sensor network systems. in 2023 *IEEE/ACIS 23rd International Conference on Computer and Information Science (ICIS)*, 55–59. (IEEE, 2023).

[40] Sundaravadivel P, Kougianos E, Mohanty SP, Ganapathiraju M (2017). Everything you wanted to know about smart health care: Evaluating the different technologies and components of the internet of things for better health. IEEE Consum. Electron. Mag..

[41] Han DM, Lim JH (2010). Design and implementation of smart home energy management systems based on zigbee. IEEE Trans. Consum. Electron..

[42] Ray PP, Dash D, Salah K, Kumar N (2020). Blockchain for IoT-based healthcare: Background, consensus, platforms, and use cases. IEEE Syst. J..

[43] Jabbar H, Song YS, Jeong T (2020). RF energy harvesting system and circuits for charging of mobile devices. IEEE Trans. Consum. Electron..

[44] Huang MW, Chen CW, Lin WC, Ke SW, Tsai CF (2017). SVM and SVM ensembles in breast cancer prediction. PLoS ONE.

[45] Gezimati, M. & Singh, G. Terahertz imaging and sensing for healthcare: Current status and future perspectives. *IEEE Access* (2023).

[46] Jiang H, Ching WK, Cheung WS, Hou W, Yin H (2017). Hadamard Kernel SVM with applications for breast cancer outcome predictions. BMC Syst. Biol..

